# Establishing a Prognostic Model Correlates to Inflammatory Response Pathways for Prostate Cancer via Multiomic Analysis of Lactylation-Related Genes

**DOI:** 10.1155/ijog/6681711

**Published:** 2025-03-21

**Authors:** Qinglong Du, CuiYu Meng, Wenchao Zhang, Li Huang, Chunlei Xue

**Affiliations:** ^1^Cheeloo College of Medicine, Shandong University, Jinan, Shandong, China; ^2^The Department of EICU, Qilu Hospital of Shandong University Dezhou Hospital, Dezhou, Shandong, China; ^3^The Department of Urology, Qilu Hospital of Shandong University Dezhou Hospital, Dezhou, Shandong, China; ^4^Center of Stem Cell and Regenerative Medicine, Gaozhou People's Hospital, Gaozhou, Guangdong, China

**Keywords:** cancer immunotherapy, lactylation, multiomics, prognostic model, prostate cancer, therapeutic drugs

## Abstract

Prostate cancer (PCa) continues to pose substantial clinical challenges, with molecular heterogeneity significantly impacting therapeutic decision-making and disease trajectories. Emerging evidence implicates protein lactylation—a novel epigenetic regulatory mechanism—in oncogenic processes, though its prognostic relevance in PCa remains underexplored. Through integrative bioinformatics interrogation of lactylation-associated molecular signatures, we established prognostic correlations using multivariable feature selection methodologies. Initial screening via differential expression analysis (limma package) coupled with Cox proportional hazards modeling revealed 11 survival-favorable regulators and 16 hazard-associated elements significantly linked to biochemical recurrence. To enhance predictive precision, ensemble machine learning frameworks were implemented, culminating in a 10-gene lactylation signature demonstrating robust discriminative capacity (concordance index = 0.738) across both primary (TCGA-PRAD) and external validation cohorts (DKFZ). Multivariable regression confirmed the lactylation score's prognostic independence, exhibiting prominent associations with clinicopathological parameters including tumor staging and metastatic potential. The developed clinical-molecular nomogram achieved superior predictive accuracy (C − index > 0.7) through the synergistic integration of biological and clinical covariates. Tumor microenvironment deconvolution uncovered distinct immunological landscapes, with high-risk stratification correlating with enriched stromal infiltration and immunosuppressive phenotypes. Pathway enrichment analyses implicated chromatin remodeling processes and cytokine-mediated inflammatory cascades as potential mechanistic drivers of prognostic divergence. Therapeutic vulnerability profiling demonstrated differential response patterns: low-risk patients exhibited enhanced immune checkpoint inhibitor responsiveness, whereas high-risk subgroups showed selective chemosensitivity to docetaxel and mitoxantrone. Functional validation in PC-3 models revealed AK5 silencing induced proapoptotic effects, suppressed metastatic potential of migration and invasion, and modulated immune checkpoint regulation through CD276 coexpression. These multimodal findings position lactylation dynamics, particularly AK5-mediated pathways, as promising therapeutic targets and stratification biomarkers in PCa management.

## 1. Background

Prostate cancer (PCa) is a major contributor to cancer-related illness and death in men globally, ranking as the second most prevalent cancer in this population [1]. Its heterogeneous nature presents challenges in diagnosis, treatment, and prognostication. Traditional methods of risk stratification often rely on histopathological features, levels of serum prostate-specific antigen (PSA), and clinical staging [2]; however, these approaches are limited in their ability to predict disease progression and treatment response accurately [3]. The era of precision oncology mandates the identification of robust molecular signatures and targetable pathways to refine PCa classification systems and therapeutic decision-making. Translational oncology research has increasingly recognized the diagnostic and therapeutic potential of post-translational modification (PTM) profiling, particularly lactylation-mediated pathway alterations in cancer progression.

Protein lactylation—a covalent modification involving lactate conjugation at lysine sites—has gained substantial recognition as a potential therapeutic target and diagnostic marker in oncology research [4, 5]. This modification is facilitated by lactate, a byproduct of glycolysis that accumulates in the tumor microenvironment, particularly in hypoxic conditions [6, 7]. Beyond its conventional role as an energy metabolite, lactate functions as a pleiotropic signaling molecule that modulates transcriptional regulation, proteostasis, and catalytic functions through diverse molecular mechanisms [8]. Emerging experimental evidence has established lactylation as a key regulatory mechanism in oncogenic signaling networks, influencing malignant transformation through modulation of proliferative signaling, apoptotic resistance, and immunosuppressive pathways [9–12]. Given the metabolic reprogramming observed in cancer cells, lactylation represents a key mechanism through which tumors adapt to their environment and promote malignancy.

In the context of PCa, the role of lactylation is particularly relevant due to the disease's association with metabolic dysregulation [13]. PCa cells often exhibit altered glycolytic activity, favoring aerobic glycolysis for establishing the characteristic Warburg metabolic phenotype through pathway dysregulation [14]. The oncogenic metabolic reprogramming serves dual functions: sustaining biosynthetic demands for uncontrolled proliferation while simultaneously reshaping the immunological landscape through inflammatory pathway activation and immune cell modulation [15]. As lactate accumulates within the tumor, its potential to modify proteins through lactylation may lead to changes in gene expression that promote tumor growth and spread. Yu et al. elucidated a novel lactylation-mediated regulatory axis in PCa progression, demonstrating that HIF-1*α* lactylation potentiates KIAA1199 expression—a hyaluronan-binding protein critical for angiogenic signaling and vascular network formation. Their pharmacological investigation identified evodiamine, a bioactive Evodiae fructus–derived compound, as a specific inhibitor of HIF-1*α* lactylation. This natural product exerts antitumor effects through dual mechanisms: induction of ferroptotic cell death and suppression of Sema3A-dependent angiogenesis while concurrently downregulating the immune checkpoint molecule PD-L1 [16, 17]. Integrative multiomic approaches leveraging high-throughput sequencing technologies and sophisticated bioinformatics pipelines have enabled systematic mapping of lactylation-regulated gene networks in diverse oncological contexts, spanning hepatobiliary, gastrointestinal, and genitourinary malignancies [18–22]. Such analyses have revealed distinct expression patterns of LRGs between tumor and normal tissues, highlighting their potential role as prognostic biomarkers. For instance, studies have indicated that certain upregulated LRGs correlate with more aggressive cancer behavior and worse prognoses, while downregulated genes may act as protective factors that confer a survival advantage to patients [23–25]. These findings imply that lactylation plays a key role in tumor biology and could be a crucial factor in determining patient prognosis.

The identification of differentially expressed LRGs in PCa has significant implications for clinical practice. By establishing a correlation between LRG expression and clinical outcomes, researchers can develop robust prognostic models that enhance risk stratification. Such models could inform treatment decisions, guiding clinicians in the selection of appropriate therapeutic strategies and monitoring patient responses [11, 12, 23, 26]. Furthermore, understanding the underlying mechanisms by which lactylation influences tumor behavior may reveal new therapeutic targets for intervention. Pharmacological inhibitors that specifically disrupt lactylation processes could offer novel avenues for PCa treatment, particularly for patients with advanced disease or those who exhibit resistance to conventional therapies [27, 28]. Beyond its prognostic significance, lactylation's influence on the tumor immune microenvironment (TIME) is gaining attention. The immune landscape around tumors is pivotal in regulating cancer progression and modulating response to therapy in PCa [29]. Mechanistic studies reveal that lactylation functions as a molecular switch governing immune cell biology, orchestrating activation thresholds, migratory capacity, and functional specialization within malignant tissues [30]. Studies have shown that lactate drives macrophage polarization toward protumor states, which may aid immune evasion and accelerate cancer growth. Furthermore, lactate also appears to establish a metabolic-epigenetic connection during this process, reinforcing immunosuppressive traits in tumor-associated immune cells [31]. Therefore, exploring the relationship between lactylation modification and TIME will help develop highly effective targeted drugs.

In our study, we underscore the importance of lactylation-related gene expression in the prognosis of PCa. By identifying key genes associated with tumor progression and establishing a robust prognostic model via multiomic analysis, we provide a foundation for future research aimed at elucidating the mechanistic roles of lactylation in cancer biology and improving treatment strategies for PCa patients. Combining single-cell and spatial transcriptomic data with detailed pathway and immune profiling underscores lactylation's significance as a pivotal focus for advancing precision oncology.

## 2. Methods and Materials

### 2.1. Source of Public Data

The Cancer Genome Atlas (TCGA)-PRAD dataset, comprising expression profiles and clinicopathological data for 488 prostate malignant samples and 50 control tissues, was acquired from TCGA database (https://portal.gdc.cancer.gov/) on August 29, 2024. For external validation, transcriptomic and clinical data from 88 PCa patients in the DFKZ-PRAD cohort were sourced from cBioPortal (https://www.cbioportal.org/). Single-cell RNA sequencing (scRNA-seq) data (GSE176031) was obtained from the Gene Expression Omnibus (GEO; https://www.ncbi.nlm.nih.gov/geo/). The IMvigor210 immunotherapy cohort was retrieved using the R package “IMvigor210CoreBiologies.” A list of 656 lactylation-associated genes was compiled from the GeneCards database (https://www.genecards.org) and existing articles [32, 33].

### 2.2. Identification of Differentially Expressed and Prognosis-Associated Lactylation Genes

The analytical protocol comprised three sequential steps: (1) extraction of 656 lactylation-associated genes from TCGA PCa transcriptomes, (2) differential expression analysis using the limma package with stringent criteria (FDR-adjusted *p* < 0.05, |log2 FC| ≥ 1), and (3) survival analysis via univariate Cox regression (*p* < 0.05) to identify disease-free survival-associated genes.

### 2.3. Establishing and Evaluating a Prognostic Model via 101 Combined Machine Learning Methods

A multialgorithm computational framework was established for PCa prognosis prediction. Ten machine learning methods were integrated: least absolute shrinkage and selection operator (LASSO), ridge regression (Ridge), elastic net (Enet), stepwise Cox regression (StepCox), survival support vector machine (survivalSVM), CoxBoost, SuperPC, plsRcox, RSF, and GBM, implemented through specialized R packages with 10-fold cross-validation [34]. The model's clinical utility was evaluated through univariate/multivariate Cox regression incorporating standard clinical parameters (age, Gleason scores, PSA values, and TNM stage). Predictive performance was quantified using time-dependent receiver operating characteristic (ROC) analysis, concordance index (C-index) calculation, and calibration curves across multiple survival endpoints (1/3/5 years), with external validation in the DFKZ dataset.

### 2.4. Building a Nomogram

To optimize predictive performance, a multivariate nomogram was constructed incorporating demographic (age), clinical staging (TNM classification), histopathological (stage), and molecular (risk score) variables. Predictive accuracy was quantified through time-dependent ROC analysis and calibration curve assessment at standardized clinical endpoints (1/3/5 years) using a specialized statistical package of time ROC.

### 2.5. Annotated Pathway Enrichment Analysis

Comprehensive functional annotation was performed through gene set enrichment analysis (GSEA) using the molecular signature profile, incorporating HALLMARK gene sets, KEGG pathways (c2.kegg.v7.4.symbols), and Gene Ontology terms (c5.go.v7.4.symbols). Differential pathway activation between risk strata was assessed using the clusterProfiler and enrichplot packages. Nine inflammation-associated pathways were extracted from MsigDB (https://www.gsea-msigdb.org/gsea/msigdb/index.jsp), with pathway activity quantification performed via single-sample gene set enrichment analysis (ssGSEA) [35]. Correlation patterns between model genes and pathway scores were evaluated using Spearman's rank correlation analysis.

### 2.6. Tumor Cell Infiltration and Tumor Mutation Burden (TMB) Analysis

Computational immunogenomic analysis was implemented using seven established algorithms (XCELL, TIMER, CIBERSORT, CIBERSORT-ABS, MCPCOUNTER, QUANTISEQ, and EPIC) for immune infiltration assessment. Immune cell composition and functional activity were profiled through ssGSEA of 16 immune subsets and 13 functional pathways. TCGA-PRAD somatic variants were processed and visualized using the maftools package.

### 2.7. Detection of LRPG Expression Level Through Multidimensional Analysis

We examined gene expression using multiple methods, including immunohistochemistry (IHC), sc-RNA-seq, and spatial transcriptomics. We retrieved the IHC data and spatial transcriptome distribution from the Human Protein Atlas (HPA, https://www.proteinatlas.org/) and the SpatialTME online database (https://www.spatialtme.yelab.site/#!/), respectively. Tumor and adjacent normal tissue sections were retrieved from Prostate_AcinarCellCarcinoma_10x_FFPE and Prostate_AdjacentNormal_10x_FFPE, respectively. The Seurat package was applied for processing data of PCa scRNA-seq data (GSE176031). The criterion for filtering cells was based on the number of detected genes (nFeature_RNA), retaining those with gene counts between 200 and 2500 and excluding cells with a mitochondrial genome proportion exceeding 5%. The FindClusters function was employed to identify cell clusters, and UMAP (Uniform Manifold Approximation and Projection) was employed to execute dimensionality reduction. Cluster-specific marker genes were identified through differential expression analysis using the FindAllMarkers algorithm, with subsequent cell type annotation performed via the CellMarker 2.0 reference database [36]. Finally, gene set scores within the single-cell RNA dataset were calculated utilizing the AUCell package.

### 2.8. Prediction of Drug Sensitivity, Potential Drugs, and Immunotherapy Response

Therapeutic response assessment was conducted through multiple computational approaches. Drug sensitivity profiles were predicted using the pRRophetic package, which calculates half-maximal inhibitory concentrations (IC50) by leveraging pharmacogenomic data from the CancerRxGene database (https://www.cancerrxgene.org/). Treatment outcomes were evaluated by comparing lactylation-related gene scores between patients exhibiting progressive disease/stable disease (PD/SD) and those achieving complete response/partial response (CR/PR) within the IMvigor210 cohort. Response rates were stratified by risk group classification. Potential therapeutic agents targeting the identified genes were identified through DSigDB database (https://www.dgidb.org/) mining, with drug-target interactions visualized using Cytoscape network analysis.

Immunotherapeutic response prediction was performed using the TIDE platform (http://tide.dfci.harvard.edu), which evaluates immune evasion potential through multiple parameters including TIDE score, CD8+ T cell infiltration, interferon-gamma signaling, immune dysfunction/exclusion indices, microsatellite instability status, and stromal cell components (CAFs, TAM M2, and MDSCs). Additionally, immunophenoscore (IPS) data from the Cancer Immunome Atlas (https://tcia.at/) were analyzed across four treatment scenarios: untreated, CTLA-4 monotherapy, PD-1 monotherapy, and combination therapy, designated as ctla4_neg_pd1_neg, ctla4_pos_pd1_neg, ctla4_neg_pd1_pos, and ctla4_pos_pd1_pos, respectively.

### 2.9. Cell Culture and Transfection

The PC-3 PCa cell line was cultivated in a specific medium with specific components. The medium consisted of 89% Ham's F-12K (Procell, #PM150901), 10% fetal bovine serum (FBS, #A567070701), and 1% Penicillin-Streptomycin Solution (Procell, #PB180120). The optimum conditions for this culture were 37°C, 95% air, and 5% CO₂. The siRNA sequences for si-NC (5⁣′-UUCUCCGAACGUGUCACGUTT-3⁣′) and si-AK5 (5⁣′-CACAGCUACACAAGAUAAATT-3⁣′) were synthesized by GenePharma (Shanghai, China) and transfected into PC-3 cells by Lipofectamine 3000 (Thermo Fisher, #L3000001) for 48 h.

### 2.10. Real-Time Quantitative Polymerase Chain Reaction (RT-qPCR)

After transfecting, PC-3 cells were harvested and lysed to extract RNA using an RNA extraction kit (EZBioscience, #EZB-RN4), followed by reverse transcription into cDNA using a commercial kit (Takara, #RR037A). The resulting cDNA, along with specific primers (AK5: forward primer 5⁣′-CTGGGCTACACTGAGCACC-3⁣′, reverse primer 5⁣′-AAGTGGTCGTTGAGGGCAATG-3⁣′) and SYBR Green (Vazyme, #Q311-02), was amplified using a thermal cycler (Thermo Fisher, 7500 Real-Time PCR System). The internal reference gene was GAPDH (forward primer 5⁣′-CTGGGCTACACTGAGCACC-3⁣′, reverse primer 5⁣′-TCTAAGCCCGAAGATCCAGTAG-3⁣′).

### 2.11. Cell Invasion and Migration in Transwell Chamber

For cell invasion analysis, Matrigel (ABW Bio, #0827045) was precoated onto the upper chamber of a 24-well transwell system (LABSELECT, #14111). For assessing the migration ability of PC-3 cells, cells were directly plated onto the upper chamber without Matrigel. Harvested cells (3 × 10^4^) were plated into the upper chamber and incubated for 48 h. A 10-min fixation with 4% paraformaldehyde was followed by a 5-min staining with 5% crystal violet on the underside of the chamber.

### 2.12. Detection of Cell Apoptosis and CD276 Expression Via Flow Cytometry

The collected cells were stained using an apoptotic cell detection kit (KeyGen Biotech, #KGA108) for 10 min and then analyzed by flow cytometry (BD Biosciences, FACScalibur). The populations of early apoptosis (annexin V(+) PI(−)) and late apoptosis (annexin V(+) PI(+)) were considered total apoptotic cells. For cell surface staining, cells were incubated with anti-CD276-APC (antigen-presenting cell) antibodies (Thermo Fisher, #MA5-15693) and isotype control (Thermo Fisher, #2324761) in the dark for 30 min, followed by flow cytometric analysis. The flow data was analyzed by FlowJo (Version 10.8.1).

### 2.13. Statistical Analysis

R program was used for data processing and visualization (v.4.2.1). To evaluate the differences between the two datasets, the Wilcoxon–Mann–Whitney *U* test was used. The log-rank test on Kaplan–Meier survival curves was used to analyze survival. Fisher's exact test was used to compare rates between the two groups, and Spearman's rank correlation coefficient was employed to determine correlations. The data was shown as mean ± SD, and a *p* value of < 0.05 was considered statistically significant.

## 3. Results

### 3.1. Prognostic Lactylation-Related Gene Expression in PCa

**Figure 1:**
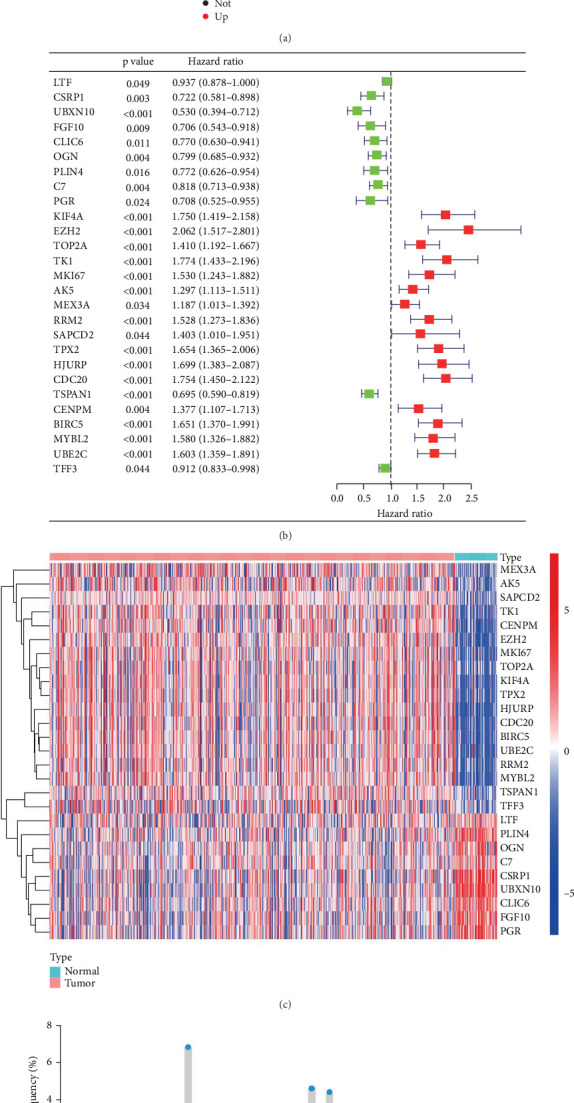
Identification of differentially expressed lactylation-related genes correlated to the prognosis of PRAD. (a) Volcano plot illustrating differential lactylation-related gene expression. (b) Forest plot showing the 27 lactylation-related genes significantly associated with PFS identified through univariate Cox regression analysis. (c) Heatmap showing the expression level of the 27 lactylation-related genes in the tumor and normal tissues. (d) Lollipop plot exhibiting the copy number alteration of the 27 lactylation-related genes.

To identify differentially expressed lactylation-related genes in PCa, we initially applied the “limma” algorithm to analyze a dataset containing 648 lactylation-related genes. Our analysis revealed significant differences in gene expression between normal and tumor tissues, with 32 genes showing upregulation and 29 showing downregulation, as illustrated in a volcano plot (Figure 1a). To further investigate the potential impact of these genes on PCa prognosis, we performed a univariate Cox regression analysis. This analysis allowed us to identify 11 protective lactylation-related genes that were associated with a better prognosis, including LTF, CSRP1, UBXN10, FGF10, CLIC6, OGN, PLIN4, C7, PGR, TSPAN1, and TFF3. Conversely, we also found 16 lactylation-related risk factors that were associated with a worse prognosis, such as KIF4A, EZH2, TOP2A, TK1, MKI67, AK5, MEX3A, PRM2, SAPCD2, TPX2, HJURP, CDC20, CENPM, BIRC5, MYBL2, and UBE2C (Figure 1b). To visualize the expression levels of these genes, we generated a heatmap that showed the distinct expression patterns of the protective and risk genes. The heatmap revealed that the 16 risk-associated genes were more highly expressed in cancer tissues compared to normal samples, while the 11 protective genes showed the opposite trend (Figure 1c). To gain additional insights into the potential role of these genes in PCa progression, we also analyzed the copy number variation (CNV) status of the lactylation-related genes. Our analysis revealed frequent alterations in 27 genes, with AK5 exhibiting the most alterations, primarily characterized by copy number losses (Figure 1d). It suggested that these genes may play important roles in PCa development and progression, and their altered expression or CNV status may contribute to the aggressive behavior of PCa. Overall, our study provides a comprehensive analysis of lactylation-related gene expression in PCa and identifies a panel of genes that may serve as prognostic indicators. These findings could be useful for predicting PCa outcomes and guiding personalized treatment strategies.

### 3.2. Prognostic PCa Model Built and Validated Using the Machine Learning Method

**Figure 2:**
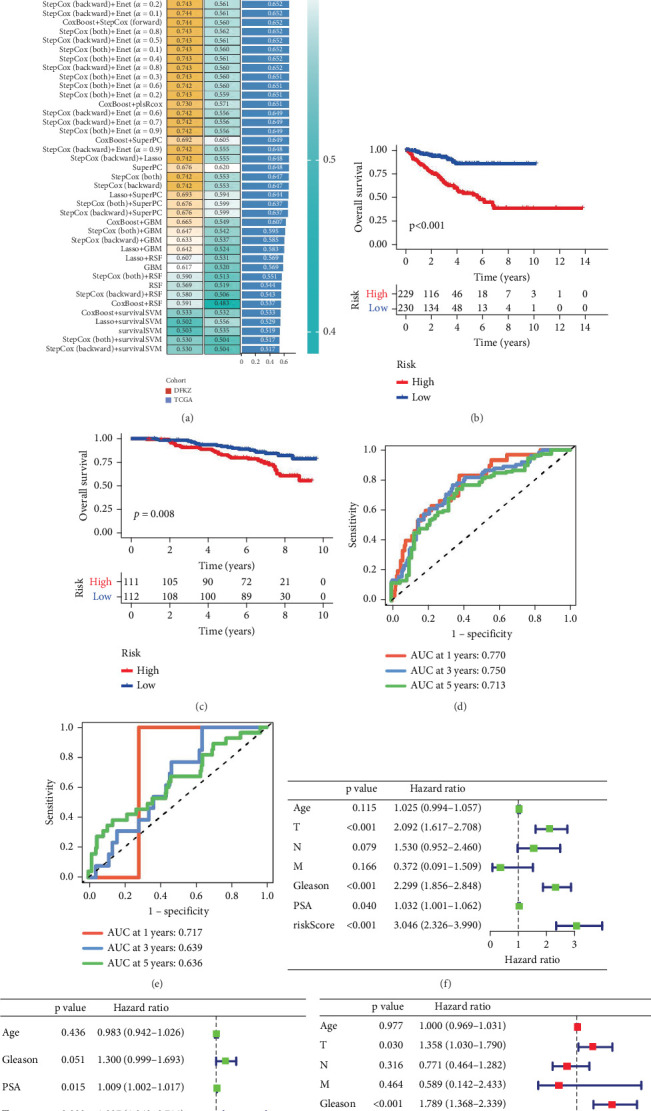
Construction of a predictive model based on the prognostic lactylation-related genes. (a) A total of 101 kinds of machine learning algorithms were applied to calculate the C-index across the TCGA (training set) and DFKZ (testing set) datasets. (b, c) Kaplan–Meier curves of PFS according to the LRPG score in TCGA and DFKZ. (d, e) ROC curves of the built model for predicting PFS at 1, 3, and 5 years. (f, g) Univariate regression was performed to determine whether the risk model was independent of other clinical features. (h, i) Multivariate regression analyses were performed to determine whether the risk model was independent of other clinical features.

To construct a robust risk model for LRPGs in PCa, we implemented an integrated machine learning framework that combined 10 distinct algorithms through a 10-fold cross-validation process. Based on the 27 candidate genes identified in our previous analysis, we employed a total of 101 algorithmic combinations to calculate the C-index for both the TCGA-PRAD cohort and an external validation dataset (DFKZ). The goal was to identify the optimal model characterized by the highest average C-index across both cohorts. Our analysis revealed that the Enet regression model with an alpha parameter of 0.9 (Enet[alpha = 0.9]) performed best, with an average C-index value of 0.738 (Figure 2a). This model included 10 genes: AK5, CDC20, CENPM, EZH2, HJURP, LTF, TFF3, TK1, TSPAN1, and UBXN10. Using the median LRPG score, we categorized PCa patients into high- and low-score groups. Patients with high-risk scores exhibited significantly reduced bRFS times in both datasets (Figure 2b,c). To further evaluate the discriminatory power of the LRPG score, we conducted a time-dependent ROC analysis. The area under the curve (AUC) values at 1, 3, and 5 years were 0.770, 0.750, and 0.713 in the TCGA cohort and 0.717, 0.639, and 0.636 in the DFKZ cohort, respectively (Figure 2d,e). These results demonstrate that the prognostic model based on the 10 LRPGs exhibits stable performance for predicting bRFS across both datasets. In summary, our study provides a comprehensive machine learning-based framework for constructing a prognostic model for PCa using LRPGs. The optimal model identified through our analysis demonstrates strong discriminatory power and may serve as a useful tool for predicting PCa outcomes and guiding personalized treatment strategies.

**Figure 3:**
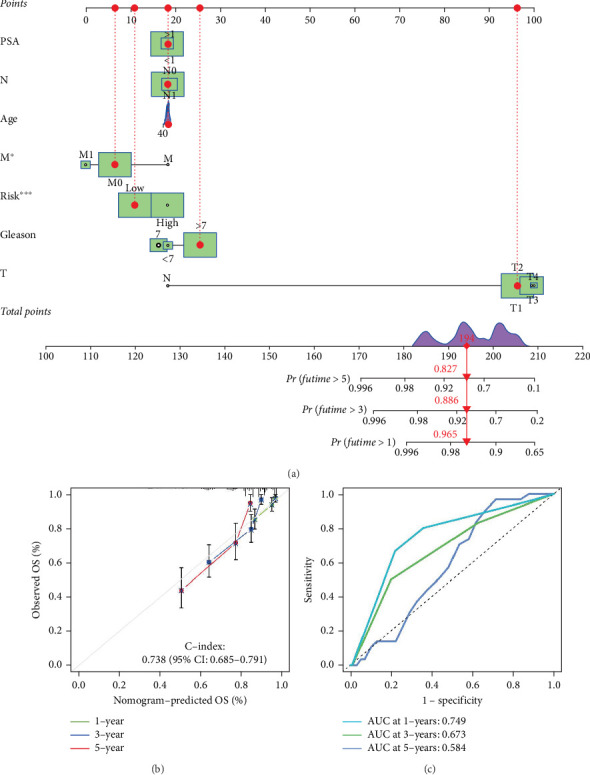
Construction of a nomogram including clinical factors and risk score for predicting bPFS of PRAD patients. (a) The nomogram integrated the risk score, PSA, age, gender, TNM stage, and Gleason score to predict bPFS. (b) The calibration curves for nomogram at 1, 3, and 5 years. (c) ROC curves assess the predicted effects at 1, 3, and 5 years.

To further evaluate the potential of the LRPG score as an independent prognostic factor for PCa, we conducted univariate and multivariate Cox analyses on our datasets. Our results indicated that both the LRPG score and tumor stage emerged as independent prognostic factors in both the training and testing datasets. Univariate Cox regression analysis suggested that the LRPG score was a significant risk factor for PCa prognosis, independent of other clinical variables such as age, TNM stage, Gleason score, and PSA levels. The hazard ratios for the LRPG score were 3.046 (95% CI: 2.326–3.990) in the TCGA cohort and 2.434 (95% CI: 1.495–3.963) in the DFKZ cohort (Figures 2f, 2g, 2h, and 2i). These findings were further supported by multivariate Cox regression analysis. We then created a nomogram predicting tool based on our Cox regression analysis that estimates bRFS for PCa patients at one, three, and 5 years by combining clinical characteristics and the LRPG score (Figure 3a). High prognostic accuracy was shown by this nomogram model, which had a C-index of 0.738 (95% CI: 0.685–0.791) (Figure 3b). To further validate the performance of our nomogram model, we conducted a time-dependent ROC analysis. The results demonstrated that the nomogram survival model performed exceptionally well in predicting multiyear survival for PCa patients (Figure 3c). Collectively, our findings underscore the clinical significance of the LRPG score as a prognostic factor for PCa and highlight the utility of the nomogram survival model in predicting outcomes for PCa patients. This nomogram model may serve as a valuable tool for guiding personalized treatment strategies and improving patient prognosis.

### 3.3. Distribution of LRPG Scores at Single-Cell and Spatial Transcriptome Levels

**Figure 4:**
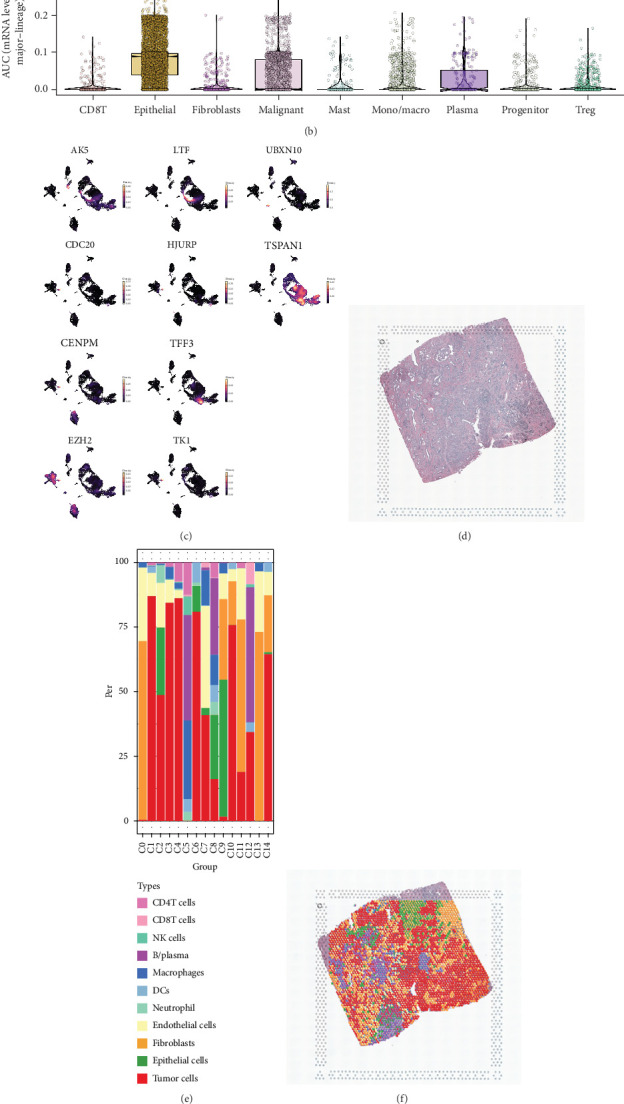
Distribution of LRPG score in the scRNA and spatial transcriptome. (a) Distribution of LRPG score in scRNA seq: UMAP of cell annotation (left) and the LRPG score was visualized by AUCell function (right). (b) Expression of AUCell scores in the different cell clusters. (c) Localization of model genes in cell populations. Distribution of LRPG score in spatial transcriptome: (d) tissue of prostate acinar cell carcinoma 10x_FFPE, (e) the proportion of cells in different clusters, (f) distribution of cell types in the tissue, and (g) distribution of LRPG score in the tissue.

To further elucidate the expression patterns of the model genes, we examined their expression and distribution from multiple perspectives. Bulk RNA-seq data from PCa were analyzed to detect the mRNA levels of the model genes, revealing that, except for LTF and UBXN10, the other genes were highly expressed in tumor tissues (Supporting Information 1: Figure S1A). We then collected IHC staining images of proteins associated with the 10 signature genes from the HPA database, which included samples from both PCa and healthy prostate tissues. Interestingly, the results for LTF protein expression were inconsistent with the mRNA findings. In contrast, the protein levels of nine model genes, excluding UBXN10, were significantly elevated in PCa tissues (Supporting Information 2: Figure S2). Next, the distribution of the model genes in various cell types was examined by searching the scRNA dataset GSE176031. As shown in Figure 4c, the expression levels of UBXN10, CDC20, TK1, HJURP, and CENPM were rarely detected, while LTF, TSPAN1, and TFF3 were mainly expressed in epithelial cells, predominantly in malignant cells. EZH2 was primarily located in monocyte/macrophage cells, whereas the expression of AK5 was detected in fibroblasts, epithelial cells, and tumor cells. Additionally, we conducted spatial transcriptome analysis to verify the heterogeneity of the spatial localization of these core genes. The analysis indicated that HJURP was barely detectable, while UBXN10 was highly expressed in adjacent paracancerous tissue sections. Conversely, other genes were highly expressed and broadly distributed in PCa sections, with TFF3 and TSPAN1 concentrated in tumor regions (Supporting Information 1: Figure S1B). These results obtained from different aspects provide us with more evidence to explore the lactation of PCa. Considering the tumor microenvironment's heterogeneity and the constraints of transcriptome sequencing in accurately reflecting gene expression, we conducted a detailed analysis of lactylation status at the cellular level by assessing the distribution of LRPG score in GSE176031 (Figure 4a). Cell annotation indicated the presence of CD8+ T cells, epithelial cells, mast cells, plasma cells, malignant cells, monocytes/macrophages, progenitor cells, and fibroblasts (Figure 4b). The results from scRNA-seq indicated that LRGP scores were present across various cell populations, predominantly in epithelial cells and subsequently in tumor cells. This suggests that lactylation activity may primarily influence these two cell types in PCa. While scRNA-seq allows for high-throughput and single-cell profiling of gene expression, it does not retain spatial information due to the dissociation of tissue during sample preparation. To examine the spatial characteristics of lactylation in the tumor microenvironment, we acquired spatial transcriptome data for PCa (Figure 4d) and subsequently clustered and annotated the cell types (Figure 4e,f).  Figure 4g illustrates that the mapping results indicated a broad distribution of LRPG scores across various cell types, consistent with single-cell findings. However, these scores were not found in all tumor cells, overlapping only with a subset of them and stromal cells.

### 3.4. Mechanism Exploration From the Perspective of Potential Pathways and Tumor Mutation

**Figure 5:**
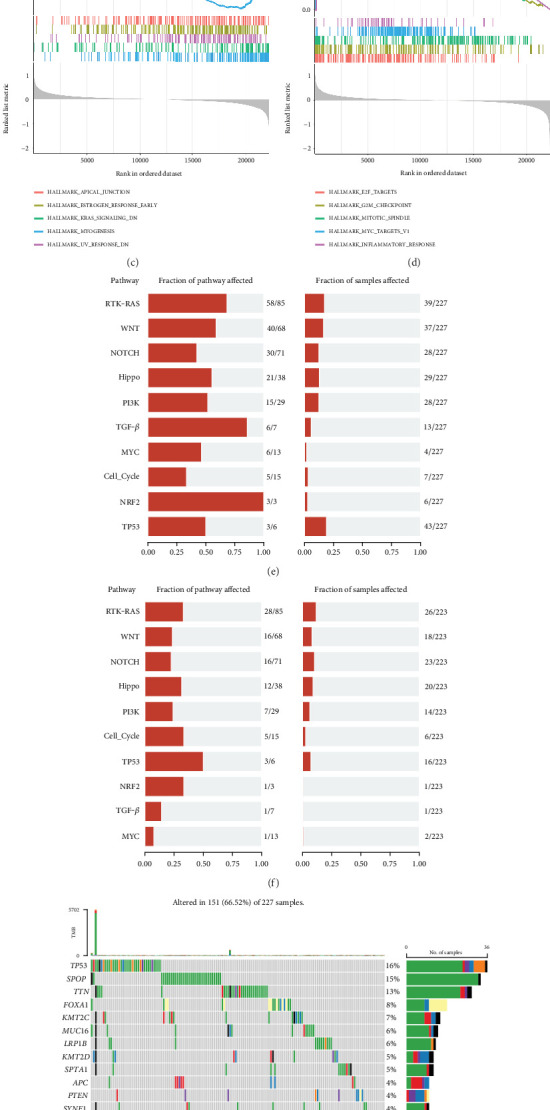
The Top 5 enriched pathways based on the GO, KEGG, and HALLMARK gene sets. (a, b) The Top 5 enriched pathways based on the GO gene sets in the two groups. (c, d) The Top 5 enriched pathways based on the KEGG gene sets in the two groups. (e, f) Fraction of oncogenic-affected pathway in the two groups. (g, h) The Top 20 mutated genes in the two groups.

To explore potential mechanisms affecting the two subgroups, three kinds of pathway enrichment analyses including GO and HALLMARK via GSEA were executed. The GO enrichment indicated that the pathways associated with muscular pathways, including GOBP_MUSCLE_CONTRACTION, GOBP_MUSCLE_SYSTEM_PROCESS, and GOBP_STRIATED_MUSCLE_CONTRACTION, were enriched (Figure 5a), while the high-risk group exhibited significant annotation in chromatin-related pathways, including GOBP_MITOTIC_SISTER_CHROMATID_SEGREGATION, GOBP_NUCLEAR_CHROMOSOME_SEGREGATION, and GOBP_REGULATION_OF_CHROMOSOME_SEGREGATION (Figure 5b). For the HALLMARK gene sets, the low-risk group exhibited enrichment in pathways such as HALLMARK_APICAL_JUNCTION, HALLMARK_MYOGENESIS, and HALLMARK_KRAS_SIGNALING_DN (Figure 5c). In contrast, the high-risk group showed enrichment in pathways including HALLMARK_E2F_TARGETS, HALLMARK_G2M_CHECKPOINT, HALLMARK_MITOTIC_SPINDLE, and HALLMARK_MYC_TARGETS (Figure 5d). GO-BP and HALLMARK's analysis revealed that the pathways associated with the inflammatory response pathway were significantly enriched in the high-risk group. Elevated lactylation levels of pyruvate kinase M2 (PKM2) have been shown to modulate the activity of proinflammatory macrophages [37]. Lactylation of Yin-Yang 1 (YY1) promotes microglial cell activation and inflammation in autoimmune uveitis [38]. Based on the findings, we investigated the association of lactylation with immune response-related pathways in PCa. The findings indicated that the low-risk group exhibited superior scores in both immune response-related pathways and the regulation of these pathways compared to the high-risk group (Supporting Information 3: Figure S3A). Correlation analysis found that LTF3 and UBXN10 were highly positively correlated with immune response-related pathways, indicating that these two lactylation genes may be the main actors regulating the linking between PCa and the response of immune function (Supporting Information 3: Figure S3B). The results suggest that the differing prognoses between the two groups are primarily attributable to the impact of chromatin-related pathways and pathways associated with the inflammatory response.

In addition, we conducted pathway and gene mutation analysis among the subgroups. The analysis indicated that oncogenic pathways, including RTK-RAS, WNT, NOTCH, and Hippo, were highly affected in the high-risk group, with a higher proportion and number of affected patients (Figure 5). Additionally, we identified the genes with the highest mutation frequency in both groups. Among PCa patients in the high-risk group, 66.52% demonstrated gene mutations (Figure 5), compared to 46.19% in the low-risk group (Figure 5). The Top 20 genes, which were not completely similar across the two groups, showed significant variations in mutation frequency. Tumor growth is linked to TP53, a tumor suppressor gene that is often altered in a variety of cancers [39]. The TP53 mutation frequency was just 4% in the low-risk group, but it was 16% in the high-risk group. With a frequency of 9% in the low-risk group and 15% in the high-risk group, SPOP was found to be the most commonly mutant gene. In line with the higher rates of mutation in tumorigenic pathways seen in high-risk patients, our results show that the frequency of gene mutations is greater in the high-risk group than in the low-risk group.

### 3.5. Differences in TIME and Immunotherapy Response

**Figure 6:**
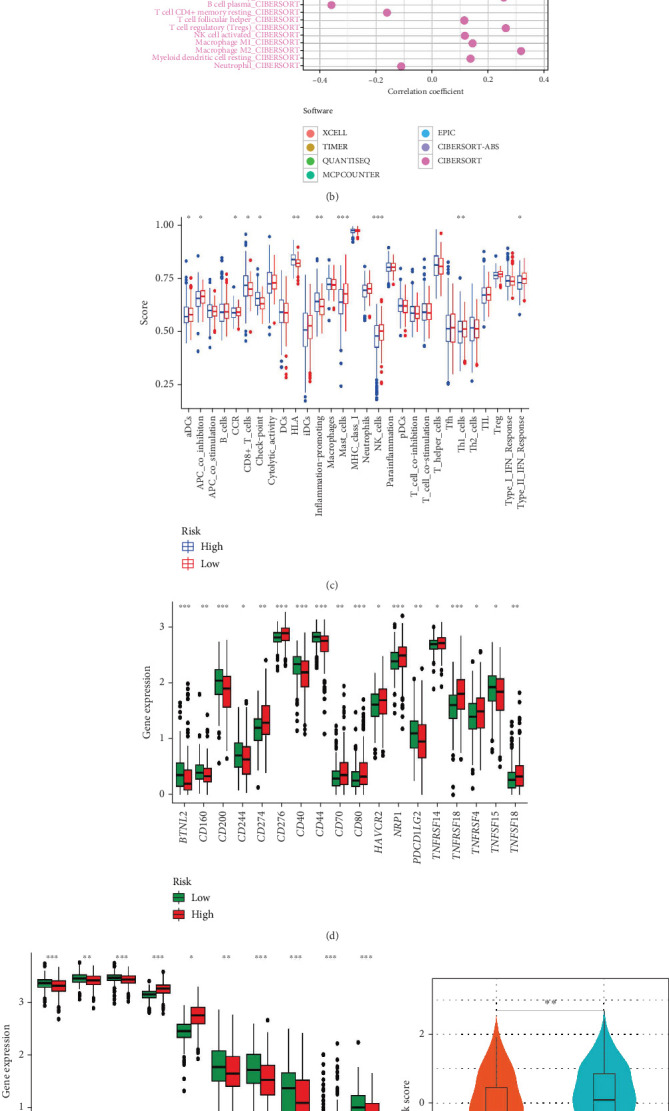
Investigation of tumor immune microenvironment and prediction of immunotherapy response. (a) Differences of stromal, immune, and ESTIMATE scores among the two groups. (b) Correlation bubble plot of the abundance of the immune cell infiltration levels with a risk score. (c) Comparison of immune-related function and immune cell infiltration between the low- and high-risk groups. (d) The differences in the checkpoint expression between the risk groups. (e) The differences in the expression of HLA genes between the risk groups. (f) Comparison of LRPG scores between the CR/PR and PD/SD patients in the IMvigor 210 cohort. (g) Percentage of CR/PR and PD/SD in the high- and low-risk patients. (h) Kaplan–Meier survival curves according to the LRPG score in the IMvigor 210 cohort.

Blood arteries, immune infiltrated cells, fibroblasts, signaling molecules, inflammatory cells coming from bone marrow, and the extracellular matrix are all part of the TIME [40]. Both the tumor and its microenvironment are intricately linked; the former may affect the latter via the release of signaling molecules, the promotion of angiogenesis, and the induction of immunological tolerance, while the latter can grow and expand tumors through the actions of immune cells [41]. Studies have shown that immune cells and stromal cells, two important types of nontumor components in the tumor microenvironment, are very valuable for tumor diagnosis and prognostic assessment [42, 43]. We delved more into the possibility that the risk signature added to TIME's complexity and variety. Immune and stromal scores were determined for both groups using the ESTIMATE score method. Based on the data, it was seen that the high-risk group had much higher immunological scores than the low-risk group, but the low-risk group also had an increased stromal score (Figure 6a). Results from a correlation study employing the XCELL method were consistent with these observations (Figure 6b). We employed seven algorithms—TIMER, CIBERSORT, CIBERSORT-ABS, QUANTISEQ, MCPCOUNTER, XCELL, and EPIC—to estimate immune cell infiltrations in PCa samples. Interestingly, the association values for B cells and macrophages were positive, but those for CD8+ T cells were negative. Additionally, we compared differences in immune cell infiltrations and functions among the two LRPG-related groups using the ssGSEA method. As depicted in Figure 6c, while CD8+ T cell numbers were lower in the high-risk group, activated dendritic cells (aDCs), mast cells, natural killer (NK) cells, and Th1 cells were more infiltrated. Additionally, we conducted a correlation study between immune cell infiltration and model gene expression. All immune cell types showed a positive correlation with LTF and UBXN10, indicating that these two genes' expression may have an impact on the TIME of PCa (Supporting Information 4: Figure S4A). Furthermore, immune-related function scores for APC coinhibition, chemokine receptor (CCR) activity, and Type II interferon response were elevated in the high-LRPG group. However, the scores for inflammation-promoting, immune checkpoint expression, and HLA presentation were higher in the low-LRPG group. We subsequently analyzed the expression levels of immune checkpoint– and HLA-related genes between the two groups, with significant differences presented in Figure 6d,e, respectively. Key immune checkpoints, such as CD274, CD276, CD80, and HAVCR2, as well as the majority of HLA genes, were significantly downregulated in the high-risk group. It suggests that the tumor immune response may be impaired in the high-risk group.

Next, using the IMvigor210 dataset, we evaluated the association between immunotherapy response and LRPG scores. Patients with PD/SD status exhibited higher LRPG scores compared to those with CR/PR status (*p* = 0.0038, Figure 6f). Notably, the percentage of patients achieving CR/PR was 17% in the high-LRPG score group, while it was up to 29% in the low score group (*p* = 2.21e − 04, Figure 6g). The low-LRPG score group showed superior prognostic results according to survival analysis (*p* = 0.0012, Figure 6h), suggesting a greater benefit from immunotherapy. Furthermore, we assessed the possibility of an immunotherapy response in PCa patients using the TIDE scoring method. The findings indicated that TIDE, dysfunction, exclusion, MSI, TAM M2, and MDSC scores were significantly higher in the high-risk group, while IFNG, MSI, and CAF scores were increased in the low-risk group (Supporting Information 4: Figure S4B). Consequently, patients in the low-LRPG group may derive greater benefit from immune checkpoint inhibitor (ICI) therapy. Furthermore, another predictive system—the IPS—yielded similar results, indicating consistently increased scores for ctla4_pos_pd1_neg, ctla4_neg_pd1_pos, and ctla4_pos_pd1_pos in the low-risk group (Supporting Information 4: Figure S4C). It seems that immunotherapy may have a substantial positive impact on individuals in the low-risk category, according to these results.

### 3.6. Differences in Chemotherapy Drug Sensitivity and Exploring Targeted Drugs

**Figure 7:**
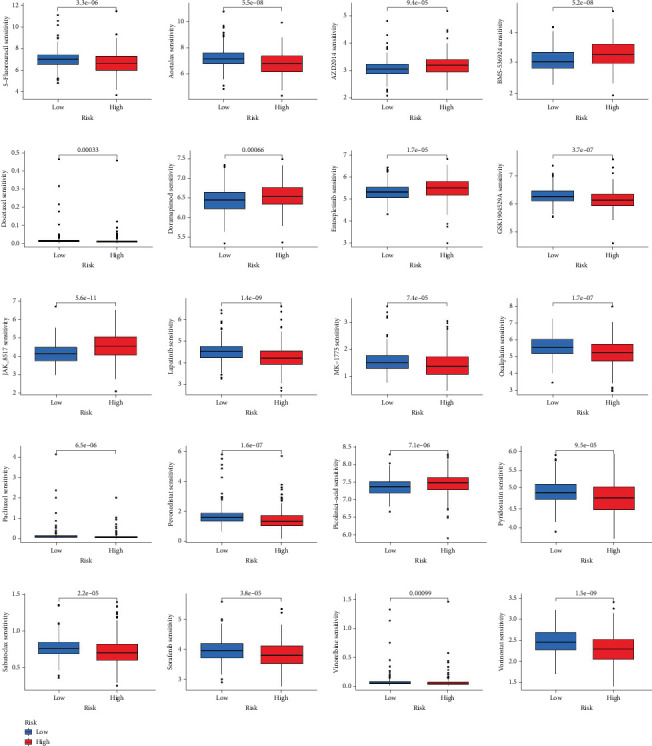
Correlation analysis of model gene expression level and drug sensitivity IC50 between the low- and high-risk groups.

We evaluated the drug sensitivity of the two groups in order to examine the model's potential for predicting the response of chemotherapeutic drugs. According to the data, the high-risk group was more sensitive to AZD2014, BMS-536924, doramapimod, entospletinib, JAK-8517, and picolinic acid, indicating that these medications could produce a better reaction in the group with higher LRPGs. In contrast, patients in the lower LRPG score group showed increased sensitivity to 5-fluorouracil, acetalax, docetaxel, GSK1904529A, lapatinib, MK-1775, paclitaxel, pevonedistat, pyridostatin, sabutoclax, sorafenib, vinorelbine, and vorinostat (Figure 7). Furthermore, we explored potential targeted drugs for the model genes and identified nine candidate drugs targeting six model genes. TSPAN1 was targeted by D-norepinephrine d-bitartrate, epinephrine, reboxetine, and sibutramine. TFF3 was targeted by ciprofloxacin, D-norepinephrine d-bitartrate, isoguanine, EINECS 250-892-2, and epinephrine, while CDC20 was targeted by hyoscyamine, bicalutamide, and thalidomide (Supporting Information 5: Figure S5).

### 3.7. Validation of Key Gene AK5 In Vitro

**Figure 8:**
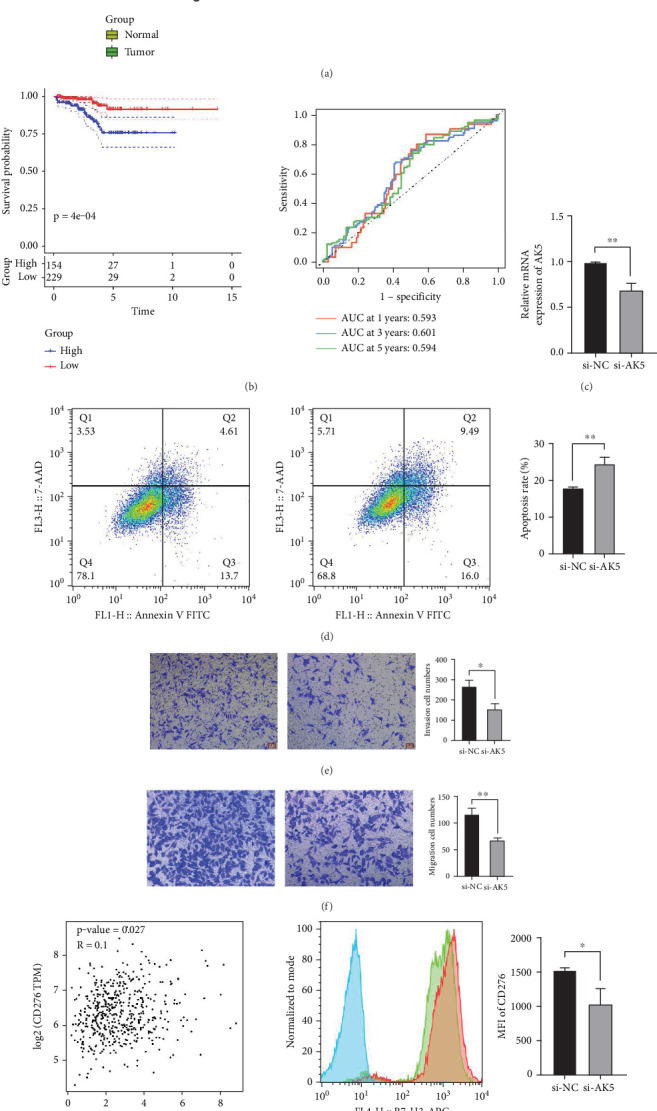
Validation of key gene AK5 in vitro. (a) Pan-cancer analysis of AK5 mRNA expression levels. (b) The prognostic value of AK5 for PRAD was determined by analyzing PFS and ROC. (c) Relative mRNA expression of AK5 treated by siRNA. (d) Apoptosis rate of PC-3 cells. (e, f) Numbers of cell invasion and migration, respectively. (g) Correlation analysis between AK5 and immune checkpoint CD276. ⁣^∗^ represents *p* < 0.05, and ⁣^∗∗^ represents *p* < 0.001.

After reviewing the literature, we found that the gene AK5 has been underexplored, with no studies specifically addressing its role in PRAD. Using TCGA pan-cancer data, we examined the expression of AK5 in tumor versus normal tissues across a variety of cancer types. The findings showed that AK5 was strongly expressed in the majority of malignancies, including head and neck squamous cell carcinoma (HNSC) and cholangiocarcinoma (CHOL) (Figure 8a). Although AK5 expression has been investigated in a pan-cancer context, its role in PRAD remains poorly understood. Therefore, we combined bioinformatics analysis with experimental validation to further investigate its potential function in PRAD. According to a bioinformatics study, progression-free survival (PFS) in PRAD was linked to AK5 expression, with a poorer prognosis being related to increased AK5 expression. Its predictive values for 1-, 3-, and 5-year survival were 0.593, 0.601, and 0.594, respectively (Figure 8b), suggesting that AK5 is a key gene linked to PRAD progression. We then successfully knocked down AK5 in the PC-3 cell line using specific siRNA (Figure 8c). This knockdown resulted in increased tumor cell apoptosis, with the apoptotic cell rate significantly higher in the si-AK5 group (17.78% ± 0.46%) compared to the si-NC group (24.69% ± 1.62%, *p* = 0.0021, Figure 8d). Furthermore, transwell assays demonstrated that AK5 knockdown significantly impaired the migratory ability of PRAD cells (*p* = 0.0112, Figure 8e). Similarly, the invasion capacity of PRAD cells was markedly reduced upon AK5 knockdown, as evidenced by the transwell assay with Matrigel results (*p* = 0.0025, Figure 8f). Furthermore, we discovered an unanticipated positive correlation between AK5 expression and the immunological checkpoint protein CD276 (B7-H3) (Figure 8g). Flow cytometry results further indicated that the downregulation of CD276 was accompanied by a significant decrease in mean fluorescence intensity (MFI) in the si-AK5 group (*p* = 0.032). These results imply that AK5 participates in tumor immunological interactions in addition to helping tumor cells behave malignantly.

## 4. Discussion

This study investigated the role of lactylation-related genes in PCa prognosis and treatment outcomes. Using univariate Cox regression, 27 lactylation-related genes were identified, and a machine learning model integrating 10 algorithms was developed to predict bRFS. The model demonstrated strong performance across datasets, with the LRPG score emerging as an independent prognostic indicator. A nomogram combining clinical features and the LRPG score provided precise survival predictions. Single-cell and spatial transcriptome analyses revealed that lactylation predominantly affects tumor cells rather than immune cells in the prostate tumor microenvironment. Pathway analysis distinguished high- and low-LRPG groups, with high-risk patients exhibiting enriched chromatin-related pathways and tumorigenic mutations. Immune profiling indicated disrupted immune cell infiltration in high-risk patients, correlating with diminished immune responses. Conversely, low-LRPG patients showed improved immunotherapy outcomes, including higher response rates to ICIs. The study also identified chemotherapy sensitivities, suggested tailored drug options for both risk groups, and analyzed the expression patterns of key model genes in PCa tissues.

PCa has the greatest frequency among male neoplasms. Although the overall survival rate for PCa is higher than for many other cancers, its mortality rate ranks second among male malignancies [44]. The risk of recurrence and metastasis in localized PCa remains substantial even after radical surgery, and these developments lead to poor patient outcomes [45]. It is urgent for researchers to explore biomarkers for identifying high-risk patients and provide insights for personalized treatment. Our study highlights the importance of lactylation-related genes in PCa prognosis and contributes to growing research on PTMs as potential cancer biomarkers. The machine learning–based risk model, constructed using the Enet[alpha = 0.9] method, demonstrated strong prognostic performance with a C-index of 0.738 across both the TCGA-PRAD and DFKZ datasets. The inclusion of genes like AK5, CDC20, and TFF3, which have established roles in cancer cell proliferation and metastasis, underscores their importance in a predictive model [46, 47]. The model's stability, confirmed through ROC analysis, supports the clinical utility of lactylation-related prognostic genes in personalized PCa risk stratification. Pan et al. constructed a model including five LRGs of ALDOA, DDX39A, H2AX, KIF2C, and RACGAP1 [20]. Compared with their model, although our model has a lower predictive power, this may be due to methodological inconsistencies, five machine learning methods, including LASSO regression analysis, random forest, XGBoost, Boruta algorithm, and SVM-RFE, while our model selected 101 combinations of 10 machine algorithms. The HR value was significantly higher than clinical factors like Gleason, PSA, TNM stage, and age, suggesting that this is an effective prediction model. The results of independent verification also showed that the model risk score was greater than 1, meaning that the higher the scores, the worse the patient's prognosis. Following external dataset verification, the model's efficacy stays constant. Prognosis may be accurately predicted by the nomogram created by integrating clinical features and LRPG scores. Nonetheless, our model performed well in patient differentiation and drug and immunotherapy response prediction.

The range of LRPG scores in PCa for different cell types revealed through scRNA-seq and spatial transcriptomics underscores lactylation's potential roles in tumor biology and the tumor microenvironment. The finding that lactylation predominantly occurs in malignant and stromal epithelial cells aligns with studies indicating that metabolic reprogramming in cancer cells can influence both tumor progression and the surrounding stromal cells [6, 7]. The minimal expression of LRPG scores in immune cells may suggest limited lactylation involvement in immune cell regulation within the tumor microenvironment of PCa, as opposed to other PTMs like acetylation, which have been more commonly linked to immune modulation [30, 48]. Functional annotation of enriched pathway analysis further supports the role of lactylation in promoting oncogenic activity within high-risk PCa patients. The chromatin-related pathways that were abundant in the high-LRPG score group, such as mitotic chromosome segregation and cell cycle processes, align with findings that lactylation may enhance transcriptional activity and chromatin accessibility, facilitating tumor proliferation [49, 50]. Conversely, low-risk patients' association with calcium signaling pathways may indicate a reduced likelihood of aggressive cellular proliferation, supporting findings in which nontumorigenic pathways correlate with better prognoses in PCa [51]. Persistent inflammation can contribute to all stages of PCa tumorigenesis, including initiation, promotion, and progression. There are many inflammatory factors that contribute to genomic instability and provide growth signals that include cytokines (such as IL-6 and TNF-*α*) and chemokines [52]. Lactylation serves as a metabolic signal linking glycolysis to epigenetic and functional changes in cells. It can regulate gene expression, cellular metabolism, and immune cell function, often in response to high lactate levels. For example, histone lactylation may upregulate proinflammatory cytokines or angiogenic factors like VEGF, linking metabolic reprogramming to a chronic inflammatory state [37]. We explored for the first time the relationship between inflammatory response-related pathways and lactylation in PCa; it provides new insights into how lactylation affects tumor progression.

The observed mutation profiles further distinguish high- and low-LRPG scores in PCa, with patients with higher scores showing increased mutation frequencies in oncogenic pathways such as the RTK-RAS, WNT, and Hippo and a higher prevalence of TP53 mutations, known to disrupt cell cycle control and tumor suppression [53–57]. Additionally, research connecting SPOP mutations to other, often less aggressive PCa subtypes is consistent with the prevalence of SPOP mutations in the low-risk population [58, 59]. With implications for therapeutic targeting of lactylation pathways in high-risk PCa patients, our results together imply that LRPGs and lactylation are key mediators of aggressive PCa phenotypes. Future research should further explore lactylation's impact on chromatin regulation and its interplay with genetic mutations to refine prognostic and therapeutic strategies. The different TIME traits and immunotherapy responses between high- and low-risk PCa groups were clarified by this research. In line with earlier research showing that a higher presence of macrophages can be associated with a worse prognosis, immune scoring using the ESTIMATE and XCELL algorithms showed that the high-LRPG score group had increased immune cell infiltration, specifically of M2 macrophages and B cells, which may contribute to an immunosuppressive environment [60–62]. Studies showing the critical function of CD8+ T cells in antitumor immunity and their frequent depletion in advanced malignancies are consistent with the decreased infiltration of these cells in patients with high LRPG scores [63, 64]. Furthermore, immune checkpoint markers and HLA genes were significantly downregulated in the high-risk group, indicating a weakened immune response, possibly hindering the efficacy of immunotherapy [65, 66]. Tumor cells, through increased glycolysis, produce high lactate levels that promote lactylation [67]. This change causes M2 macrophages to become more polarized, which in turn creates an immunosuppressive tumor microenvironment that reduces the effectiveness of ICIs such as PD-1/PD-L1 and CTLA-4 blockers [68]. Histone lactylation also affects gene expression, enhancing pathways that support immune evasion and reducing T cell infiltration in the TME. This contributes to T cell exhaustion and dysfunction, both of which limit antitumor responses crucial for ICIs [5, 69]. Consequently, tumors with high lactylation levels often demonstrate resistance to immunotherapy. Targeting lactate production or export—through lactate dehydrogenase or monocarboxylate transporter inhibitors—could reduce lactylation, potentially reversing immune suppression and enhancing ICI efficacy [70]. Moreover, combining ICIs with epigenetic modulators, such as HDAC inhibitors, may counteract lactylation-induced gene expression changes, promoting immune cell activity [71]. In several types of cancer, CD276 is often overexpressed and mainly affects T cell responses. It has been shown to inhibit T cell growth and activation, which helps malignancies evade the immune system [72]. We conducted a correlation analysis between AK5 and several immune checkpoint molecules typically expressed on cancer cells and/or APCs, such as PD-L1, CD155, and GAL-9 [73]. Our analysis revealed a positive correlation between AK5 and CD276. The downregulation of CD276 following a reduction in AK5 expression in tumor cells suggests that targeting AK5 may enhance the efficacy of immunotherapies that leverage the CD276 checkpoint strategy. Overall, lactylation may serve as both a predictive biomarker for immunotherapy response and a promising therapeutic target, where reducing lactylation could enhance outcomes for patients with immunotherapy-resistant tumors. This study also provides valuable insights into advancing personalized immunotherapy approaches.

Notwithstanding the positive findings, this research has many drawbacks. First, even though the research included many datasets, such as the TCGA and DFKZ cohorts, we still need to gather and create other, bigger datasets for outside validation in order to verify that the results are broadly applicable. Secondly, prognosis prediction using machine learning models has its limitations, such as the likelihood of bias in the training set and the reliance on the quantity and quality of the available data. Third, even though this study focused on gene clusters linked to lactylation, the mechanistic knowledge of lactylation in PCa and its effect on PCa is still lacking since the subject is still in its infancy and the function of lactylation in cancer biology is not well known. In addition, while single-cell and spatial transcriptome analyses provide valuable insights into the distribution of lactylation activity in PCa, they rely on data from existing databases that may not fully capture the heterogeneity or spatial complexity of tumors, especially spatial transcriptome analyses, which only analyzed one slice. Immune profiling, while informative, could benefit from a deeper exploration of the dynamic interactions of the immune microenvironment and how it promotes tumor progression. Lastly, in order to assess the effectiveness of these medications in clinical practice, experimental validation via in vitro and in vivo investigations is required, even if possible therapeutic targets and drug sensitivities have been discovered.

## Data Availability

The internet address provided in the Methods and Materials section provides access to the public datasets gathered for this research.

## Supporting Information

Supporting Information 1 Figure S1: Multilevel analysis of model gene expression. (a) Expression mRNA levels of model genes in PCa. (b) Expression levels of model genes in PCa tissues validated by spatial transcriptome.

Supporting Information 2 Figure S2: Expression levels of model genes in PRAD tissues validated by IHC.

Supporting Information 3 Figure S3: Correlation analysis between inflammatory response–related pathways and scores. (a) Differences in inflammatory response–related pathways between the two groups. (b) Correlation analysis between model genes and inflammatory response–related pathways.

Supporting Information 4 Figure S4: Prediction of immunotherapy response via TIDE and IPS scoring system. (a) Correlation analysis between model genes and immune cell infiltration. (b) Differences in immune-related scores among the two risk groups were estimated by the TIDE method. (c) Differences in IPS among the two risk groups were estimated by the IPS method.

Supporting Information 5 Figure S5: Potential drugs targeted to the model genes. The circles represent the significance of the relationship between the drug and the gene; the larger, the greater the significance.
