# Supplementary figures and images for: Establishing a Prognostic Model Correlates to Inflammatory Response Pathways for Prostate Cancer via Multiomic Analysis of Lactylation-Related Genes
Source: Int J Genomics. 2025 Mar 21;2025:6681711. doi: 10.1155/ijog/6681711 (PMC11952923; doi:10.1155/ijog/6681711)

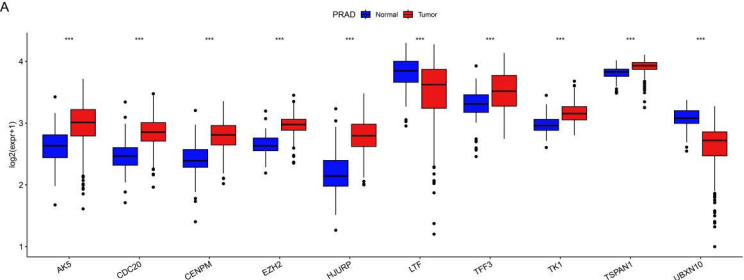

B

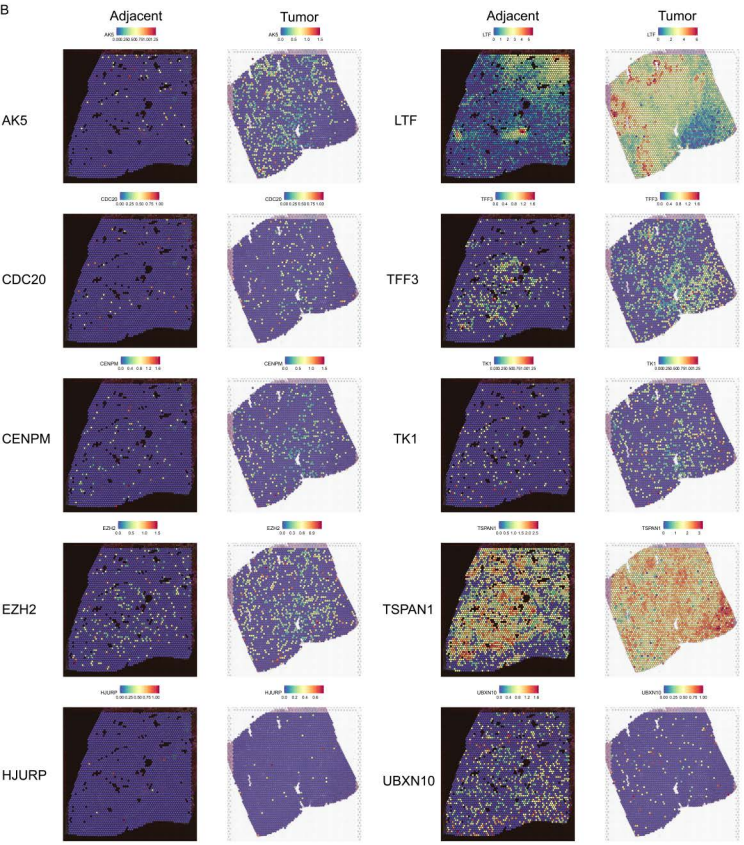

Supplement: Supporting Information 1 — Figure S1: Multilevel analysis of model gene expression. (a) Expression mRNA levels of model genes in PCa. (b) Expression levels of model genes in PCa tissues validated by spatial transcriptome. [file 6681711.f1.pdf]

Normal

Tumor

Normal

Tumor

AK5

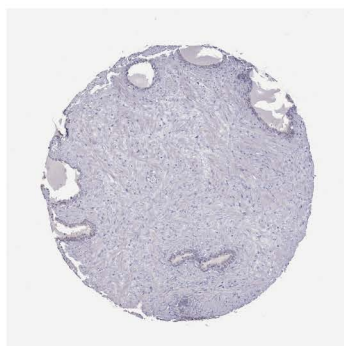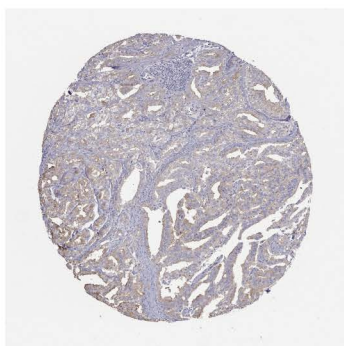

LTF

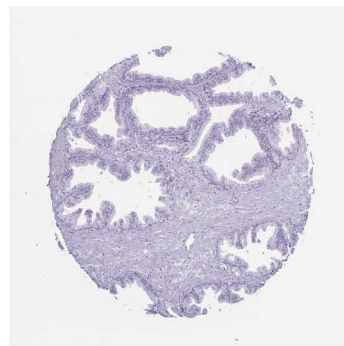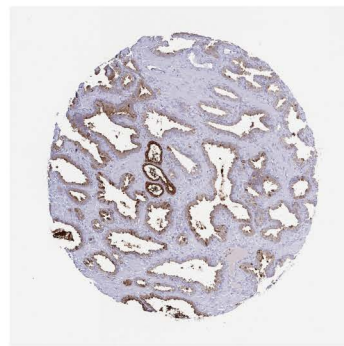

CDC20

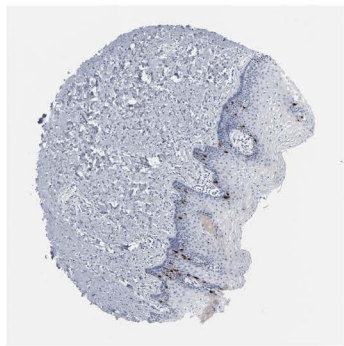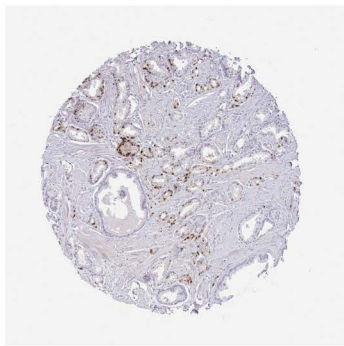

TFF3

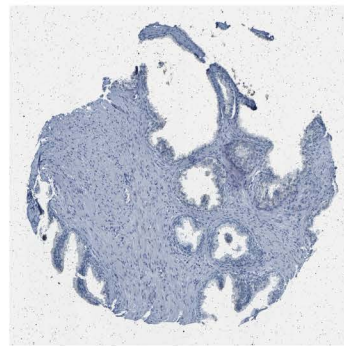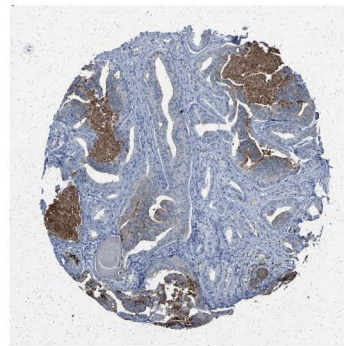

CENPM

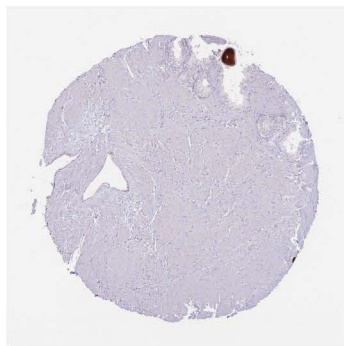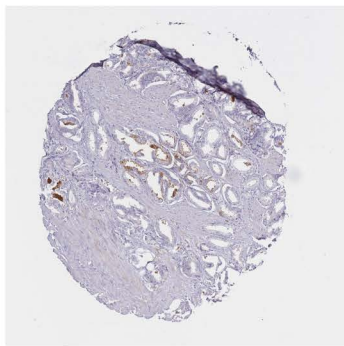

TK1

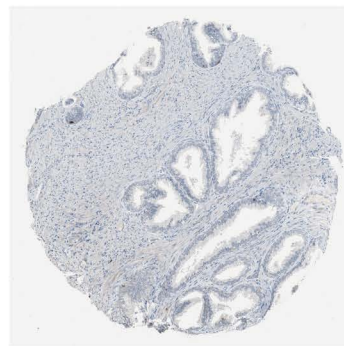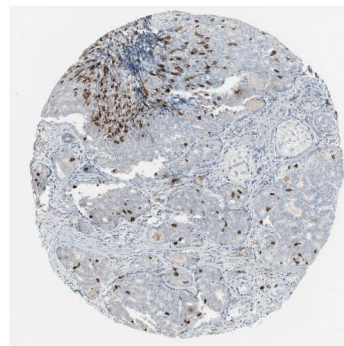

EZH2

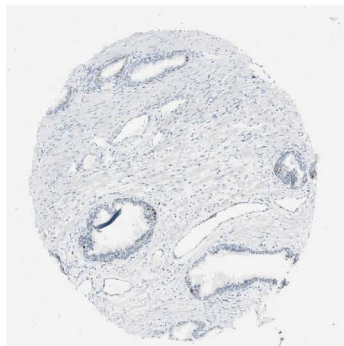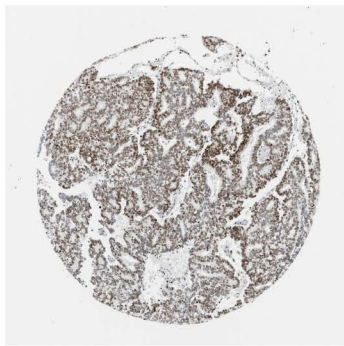

TSPAN1

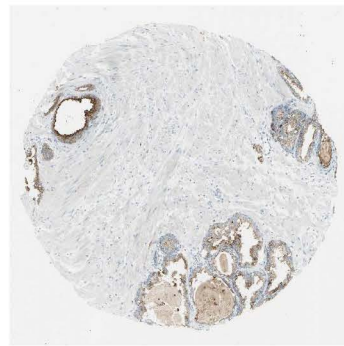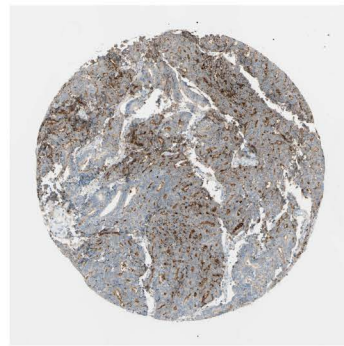

HJURP

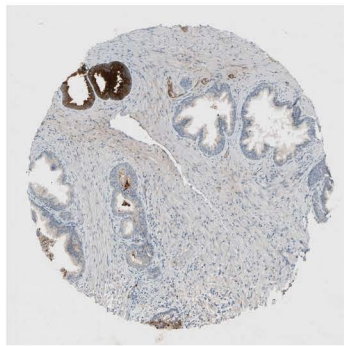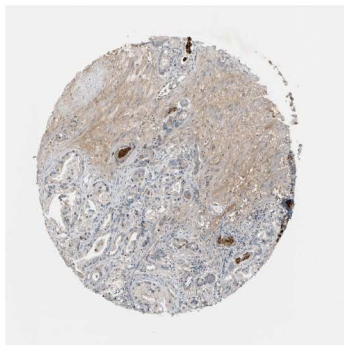

UBXN10

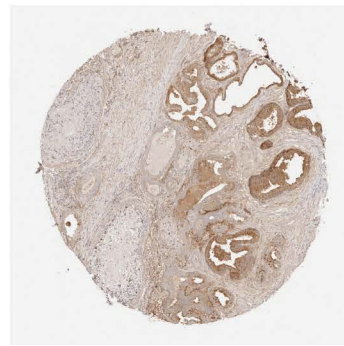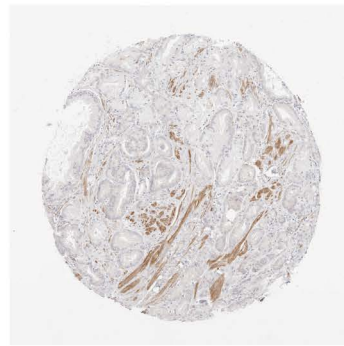

Supplement: Supporting Information 2 — Figure S2: Expression levels of model genes in PRAD tissues validated by IHC. [file 6681711.f2.pdf]

A

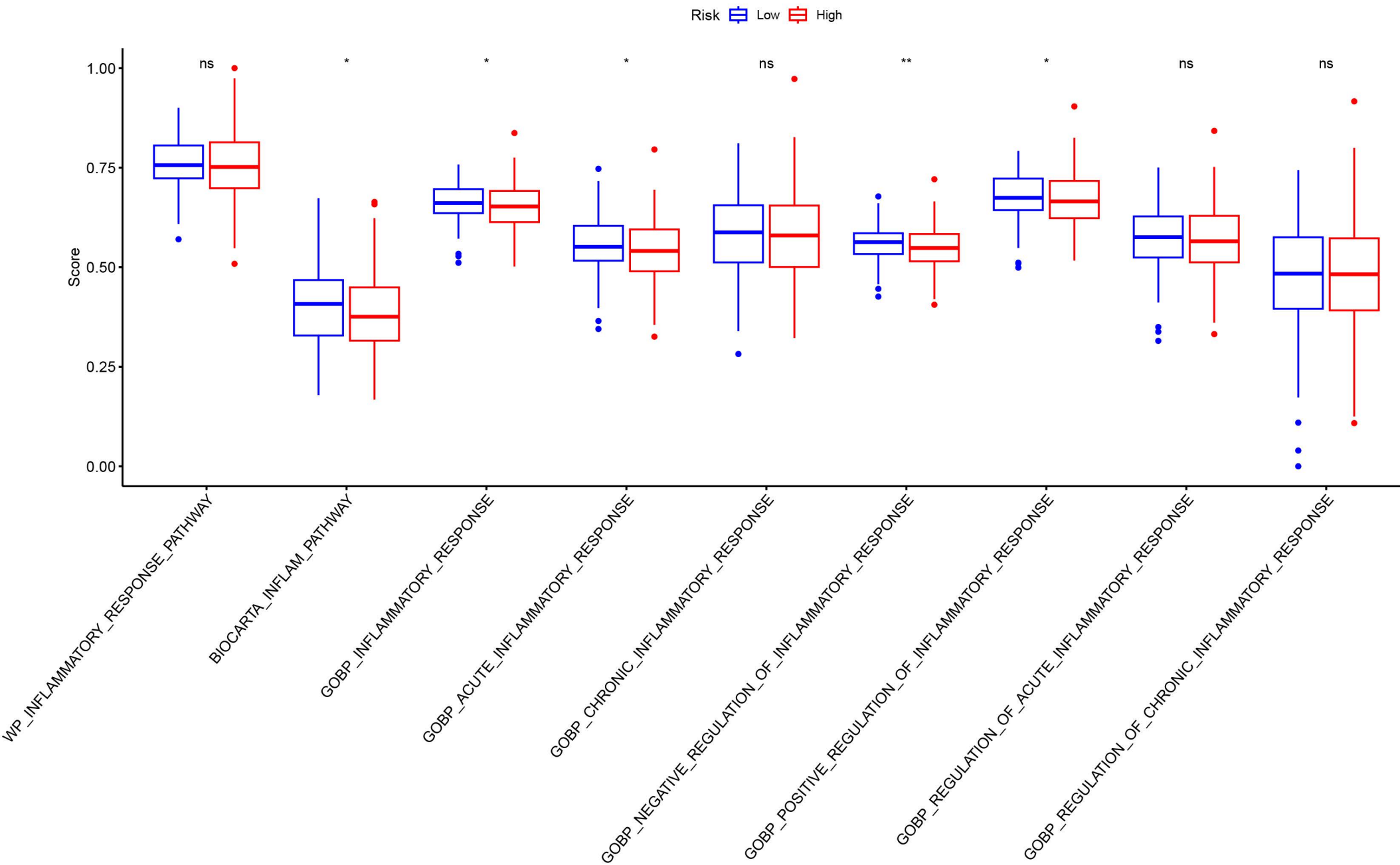

B

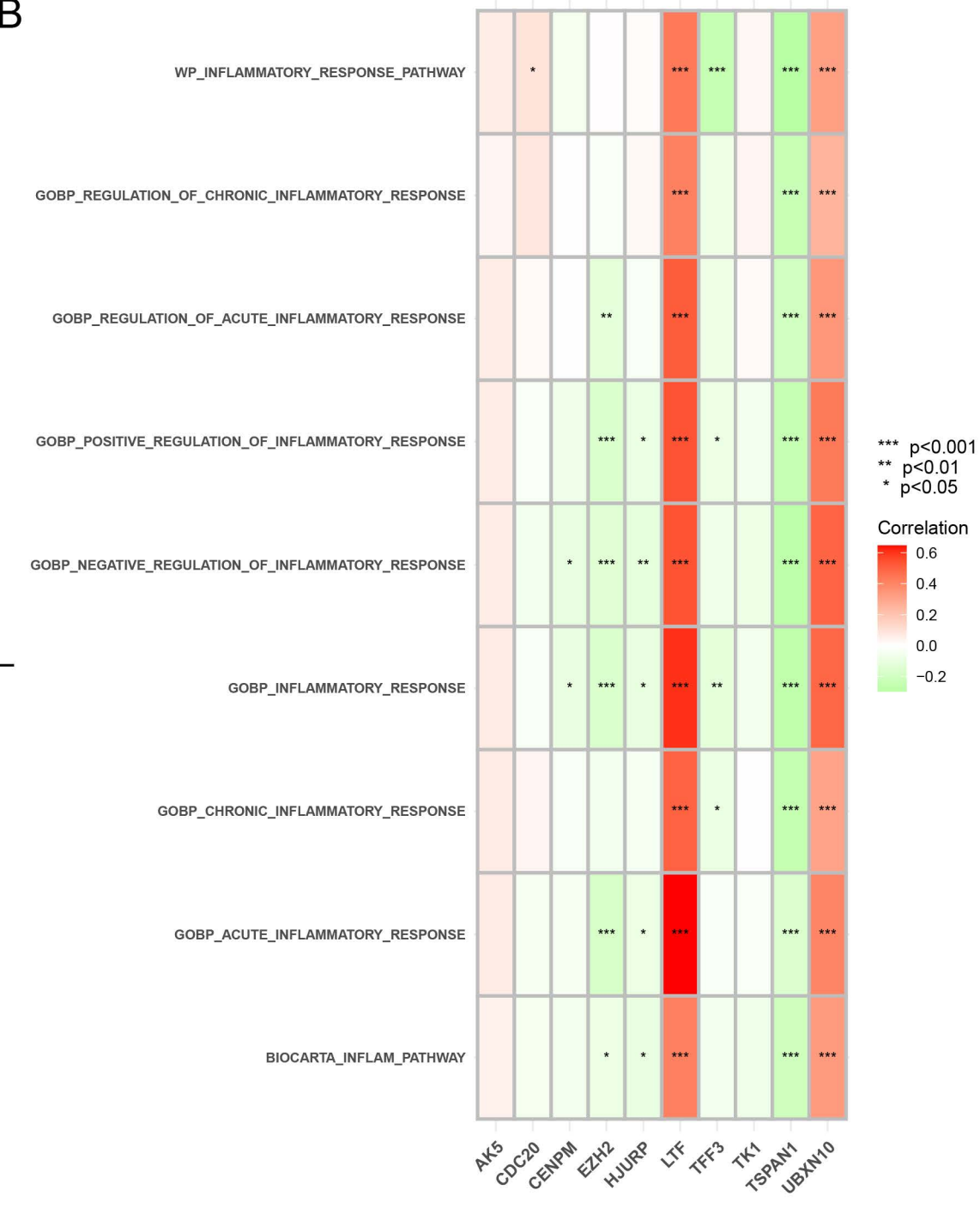

Supplement: Supporting Information 3 — Figure S3: Correlation analysis between inflammatory response–related pathways and scores. (a) Differences in inflammatory response–related pathways between the two groups. (b) Correlation analysis between model genes and inflammatory response–related pathways. [file 6681711.f3.pdf]

A

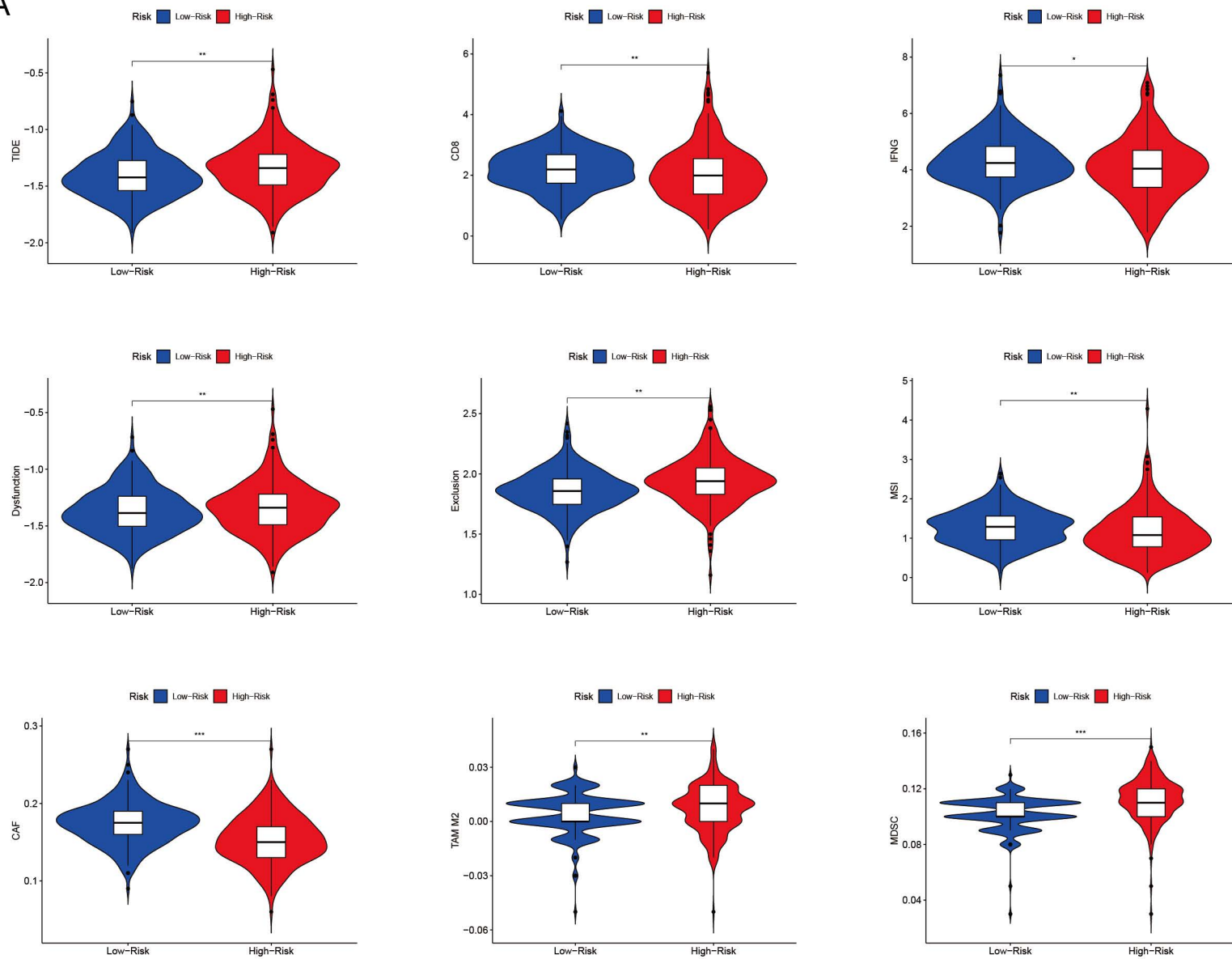

B

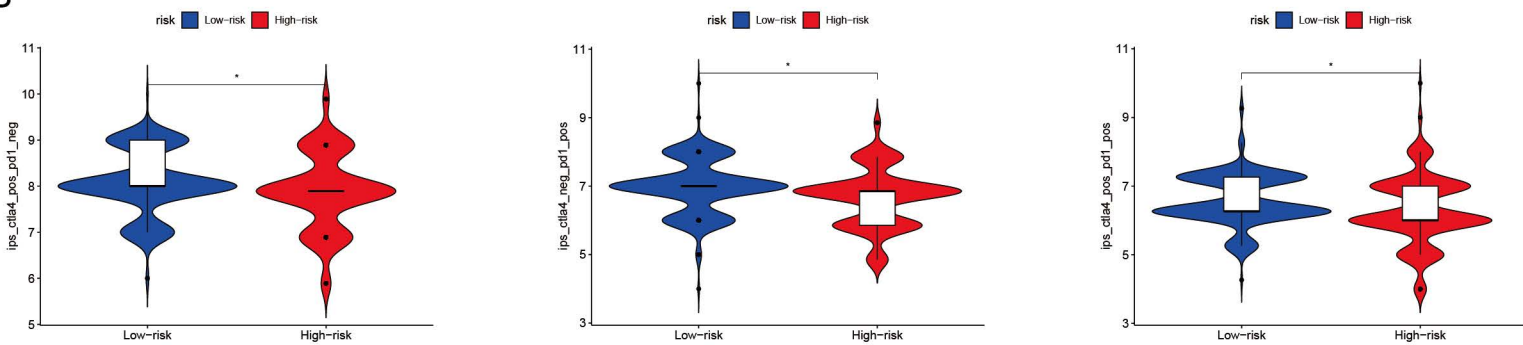

Supplement: Supporting Information 4 — Figure S4: Prediction of immunotherapy response via TIDE and IPS scoring system. (a) Correlation analysis between model genes and immune cell infiltration. (b) Differences in immune-related scores among the two risk groups were estimated by the TIDE method. (c) Differences in IPS among the two risk groups were estimated by the IPS method. [file 6681711.f4.pdf]

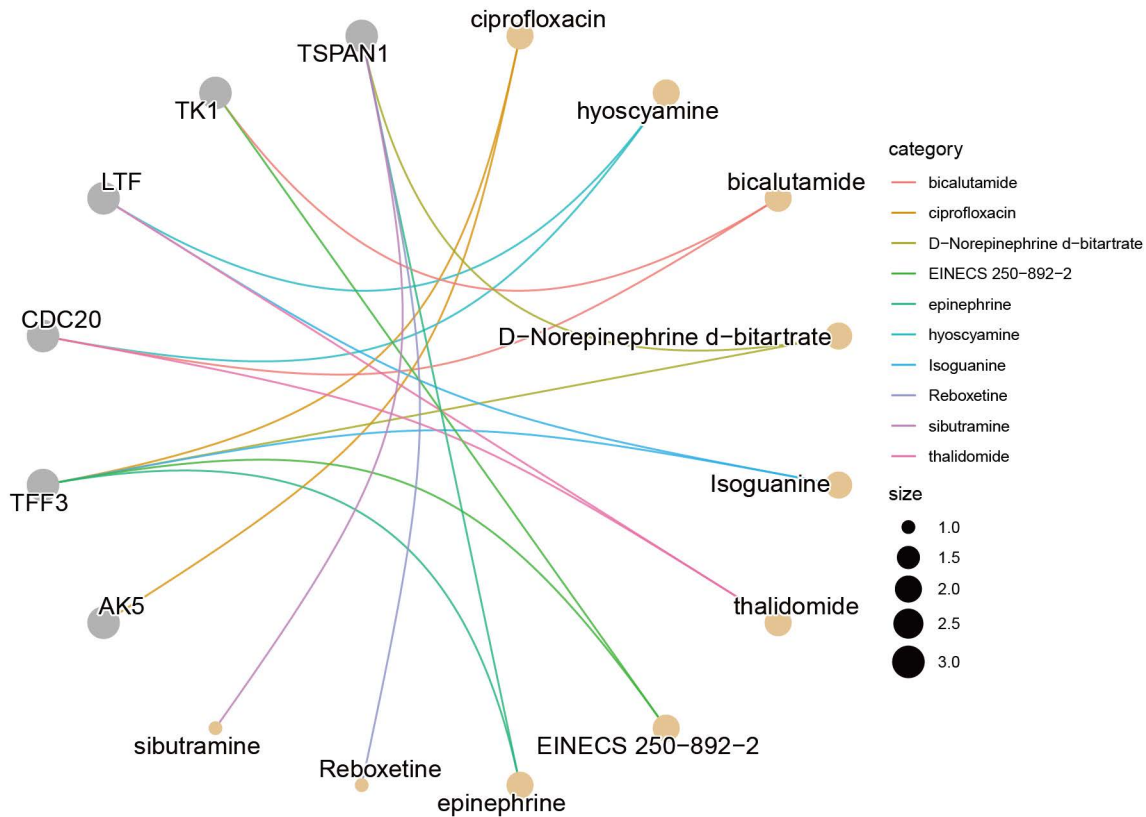

Supplement: Supporting Information 5 — Figure S5: Potential drugs targeted to the model genes. The circles represent the significance of the relationship between the drug and the gene; the larger, the greater the significance. [file 6681711.f5.pdf]
